# Data-driven cranial suture growth model enables predicting phenotypes of craniosynostosis

**DOI:** 10.1038/s41598-023-47622-7

**Published:** 2023-11-23

**Authors:** Jiawei Liu, Joseph H. Froelicher, Brooke French, Marius George Linguraru, Antonio R. Porras

**Affiliations:** 1 Department of Biostatistics and Informatics, Colorado School of Public Health, University of Colorado Anschutz Medical Campus, Aurora, CO 80045 USA; 2 Department of Pediatric Plastic and Reconstructive Surgery, Children’s Hospital Colorado, Aurora, CO 80045 USA; 3 Department of Surgery, School of Medicine, University of Colorado Anschutz Medical Campus, Aurora, CO 80045 USA; 4 Sheikh Zayed Institute for Pediatric Surgical Innovation, Children’s National Hospital, Washington, DC 20010 USA; 5 Departments of Radiology and Pediatrics, George Washington University School of Medicine and Health Sciences, Washington, DC 20052 USA; 6 Department of Pediatric Neurosurgery, Children’s Hospital Colorado, Aurora, CO 80045 USA; 7 Departments of Pediatrics and Biomedical Informatics, School of Medicine, University of Colorado Anschutz Medical Campus, Aurora, CO 80045 USA

**Keywords:** Computational biology and bioinformatics, Computational models, Image processing, Machine learning, Statistical methods, Musculoskeletal system

## Abstract

We present the first data-driven pediatric model that explains cranial sutural growth in the pediatric population. We segmented the cranial bones in the neurocranium from the cross-sectional CT images of 2068 normative subjects (age 0–10 years), and we used a 2D manifold-based cranial representation to establish local anatomical correspondences between subjects guided by the location of the cranial sutures. We designed a diffeomorphic spatiotemporal model of cranial bone development as a function of local sutural growth rates, and we inferred its parameters statistically from our cross-sectional dataset. We used the constructed model to predict growth for 51 independent normative patients who had longitudinal images. Moreover, we used our model to simulate the phenotypes of single suture craniosynostosis, which we compared to the observations from 212 patients. We also evaluated the accuracy predicting personalized cranial growth for 10 patients with craniosynostosis who had pre-surgical longitudinal images. Unlike existing statistical and simulation methods, our model was inferred from real image observations, explains cranial bone expansion and displacement as a consequence of sutural growth and it can simulate craniosynostosis. This pediatric cranial suture growth model constitutes a necessary tool to study abnormal development in the presence of cranial suture pathology.

## Introduction

The neonatal cranium is formed by different bone plates separated by fibrous tissue called sutures. This structure allows the cranial bones to displace and expand to create space for a rapidly growing brain^1^. When the brain does not need additional volume, the cranial bone plates fuse at the sutures. Unfortunately, 1 in 2100 live births present a condition called craniosynostosis in which one or more cranial sutures fuse early^2^. Patients with this condition typically present with brain growth constraints perpendicular to the fused sutures and cranial malformations^3^. Surgical treatment is usually indicated for these patients to remove their growth constraints and treat their aesthetic malformations^4^. However, since there are no personalized tools to study and predict development in these patients, the adequate time for treatment and the surgical approach are highly variable and subjective^5–8^.

Personalized developmental predictions have traditionally been challenging because of the lack of quantitative information about the rates of sutural growth and bone displacement in children. Consequently, there is limited understanding of the abnormal development of patients with cranial bone and suture pathology such as craniosynostosis. These limitations are important contributing factors to the uncertainty about the potential causes of this condition, which are unknown in more than 85% of children with craniosynostosis^9, 10^.

Existing reports on longitudinal cranial growth provides descriptive statistics of simple cranial metrics (such as head circumference, width, and length) on discrete time points^11, 12^, which are not predictive of local cranial development. Most existing works modeling cranial growth can be grouped into simulation-based and statistical methods. Simulation-based methods hypothesize biomechanical properties of the cranial bones and growth rates to make temporal predictions given a cranial geometry, either synthetic or obtained from images. For example, finite element modeling (FEM) has been used to study suture morphology and sutural collagen fibers response to tensile loads^13^, and to better understand the biomechanics of the cranial sutures in other mammals^14, 15^. Other FEM models were used to predict craniofacial bone strains by measuring displacements of a tensile loaded in-vitro mandible, comparing measured and predicted strains^16^, and to predict blunt- and ballistic impact-related skull and pressure-related brain injuries^17^. Hindered by the lack of population-based data to build and validate simulations, the main limitation of these methods is their simplifications and assumptions about the biophysical processes driving development. For example, Libby et al.^18^ created two FEMs to predict cranial displacements during the first year of life based on one skull from a cadaver. Then, they compared one FEM to a 3d-printed in-vitro physical model and the other to computed tomography (CT) images of 56 different subjects in terms of global shape and volume.

Unlike simulation-based approaches, statistical methods leverage existing datasets to infer statistically the anatomical variability observed in the population. Li et al. used principal components analysis (PCA) and temporal regression to build independent models of cranial growth using 56 CT images of children aged 0–3 year^19^ and 42 CT images of children aged 3–10 years^20^, respectively. Removing linearity constraints and accounting for sex, our group created a complete data-driven normative reference for cranial development during the first 10 years of life using a large cross-sectional CT image dataset of 2068 subjects^21^. However, given the lack of longitudinal image datasets, these methods could not provide specific information about sutural growth and predictions were not personalized to specific patient anatomies. Partly addressing these limitations, our group also built a personalized spatiotemporal cranial growth model using 278 cross-sectional CT images based on a multi-resolution bilinear PCA method to model both anatomical variability in the population and temporal changes^22^. However, this personalized predictive model still could not infer sutural growth rates to explain the observed anatomical changes in the population. Although other temporal shape-based regression methods^23,24^ have also been extensively studied to model temporal changes in other anatomical structures, these models struggle to interpret the local processes driving anatomical changes or to simulate pathology.

This work quantifies suture growth rates in humans for the first time by presenting a novel cranial development model explicitly explained by sutural growth. Our model was designed to address the main limitations of simulation-based approaches (based on assumptions not fully supported by data), and of traditional statistical methods (limited interpretability and inability to predict pathological growth). Our group built on the concepts from our previous locally affine shape registration framework^25^ to design a diffeomorphic cranial development model where bone plate expansion and displacement are a consequence of bone growth at the cranial sutures. To build our model, our group used the cranial segmentations from a cross-sectional CT image dataset of subjects without cranial pathology and inferred statistically local sutural growth rates between birth and 10 years of age. Then, our group used our model to predict normative cranial growth. In addition, our group modified it to predict cranial growth in the presence of craniosynostosis. Our group evaluated the model’s predictive accuracy using independent normative and pathologic longitudinal datasets.

## Materials and methods

### Data description

**Figure 1:**
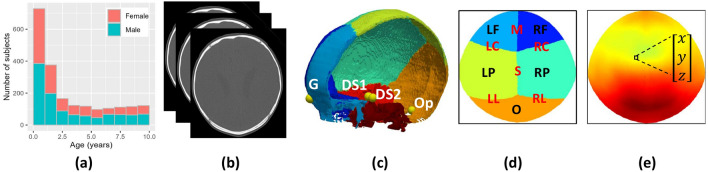
Data distribution and CT image processing pipeline. (**a**) shows the age distribution of the normative population in Dataset A. (**b**) shows an example of 3D volumetric CT. (**c**) shows the location of the cranial base landmarks, where G, DS1, DS2 and Op represent the glabella, two clinoid processes of the dorsum sellae and opisthion, respectively. (**d**) represents the cranial bone labels color-coded in a standardized 2D spherical map, where LF, RF, LP, RP and O represent left and right frontal, left and right parietal, and occipital bones, and M, LC, RC, S, LL, and RL represent metopic, left coronal, right coronal, sagittal, left lambdoid, and right lambdoid sutures, respectively. (**e**) shows the representation of the Euclidean coordinates of every point in the calvaria using a standardized 2D spherical map. The color represents the magnitude of the Euclidean coordinate vector at every point.

Informed consent was waived by the Institutional Review Boards (IRB) of the University of Colorado (protocol #20-1563) and Children’s National Hospital (protocol #3792) for this secondary research study. Four retrospective datasets of patients younger than 10 years were collected after obtaining IRB approval and all methods were performed in accordance with the relevant guidelines and regulations. Dataset A contains retrospective CT images of 2068 normative subjects (965 female) with age 3.12 ± 3.05 years (see age distribution in Fig. 1a) and was used to construct the model. A higher number of subjects in early ages enables us to capture faster cranial changes in early life^26^. These subjects were referred to the emergency room for trauma and clinical evaluation discarded any cranial anomaly as described in previous work^21^. Dataset B contains two longitudinal CT images from each of 51 normative subjects (23 female, 2.24 ± 2.22 years at the first image and 3.55 ± 2.71 years at the second image) and was used to evaluate the accuracy of the model predicting normal growth. Datasets C contains cross-sectional CT images of 212 patients (70 female) with single suture craniosynostosis (31 metopic, 133 sagittal and 48 unicoronal) with age 0.59 ± 1.02 years. Finally, Dataset D contains 14 longitudinal image pairs. Specifically, 10 image pairs were available from 10 patients (4 female) with single suture craniosynostosis (2 metopic, 5 sagittal and 3 unicoronal), with age of 0.61 ± 0.60 at the first image and 1.24 ± 1.07 at the second image. In addition, two patients in Dataset D had a third CT image acquired at an average age of 3.04 ± 2.23 years. Hence, four additional image pairs were available by pairing the first available image with the third, and the second image with the third for those two patients. Datasets C and D were used to evaluate the accuracy of our model simulating craniosynostosis. The average in-plane image resolution of the CT images across datasets was 0.37 ± 0.06 mm with slice thickness of 1.97 ± 1.28 mm.

### CT image processing

We used our previous methods to automatically extract relevant information from the CT images as depicted in Fig. 1b^22, 27^. In summary, we used adaptive thresholding^28^ to segment the skull from CT image as shown in Fig. 1c. We used our deep learning model^27^ to detect the cranial landmarks^29^ and label the cranial bone plates as shown in Fig. 1c. Any inaccuracies in bone labeling or landmark location were manually corrected. The segmented images were transformed to volumetric meshes using marching cubes algorithm^30^ and sampled in spherical coordinates to create simplified two-dimensional (2D) manifold-based representations, which were iteratively registered using the diffeomorphic demons algorithm^31^ to establish local correspondences between subjects guided by the location of the cranial sutures as proposed in^22^. After this process, the calvarial surfaces of all subjects were represented using a standard spherical representation with point correspondences between subjects and uniform pose. Figure 1d, e show examples of the 2D spherical map representations for one patient.

### Diffeomorphic cranial development model

**Figure 2:**
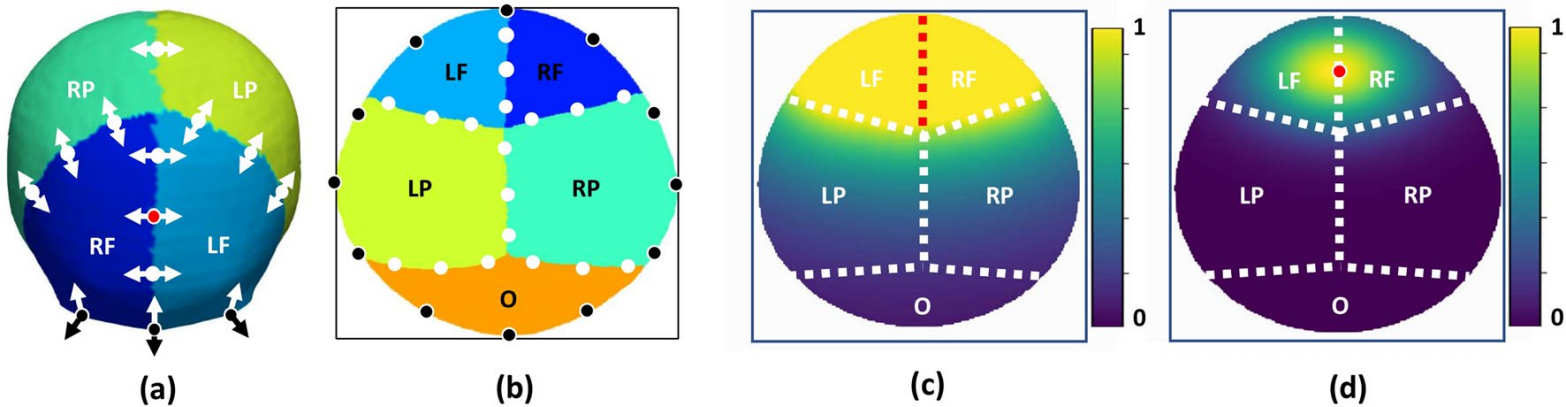
Suture growth directions and spatial weight functions. (**a**) shows the direction ${{\varvec{u}}}_{{\varvec{a}}}$ (as white arrows) of bone growth tangential to the cranial surface and perpendicular to the suture at different suture locations, and direction ${{\varvec{y}}}_{{\varvec{a}}}$ (as black arrows) perpendicular to the cranial surface at the cranial base. (**b**) shows the uniformly discretized control points at each suture in our model. (**c**) Representation of the spatial weight function ${w}_{l}$ associated with the metopic suture. (**d**) Representation of the local weight function ${w}_{l}^{a}$ associated with one control point (in red) in the metopic suture.

We propose a spatiotemporal transformation model that explains the anatomical changes at every point on the cranial surface as a consequence of the local bone growth at the sutures perpendicular to them^3^. In addition, our model incorporates the local displacements produced at the cranial base to account for the growth of the facial and cranial base structures, which adjust the position of the calvaria. Figure 2a represents the directions of sutural growth and cranial base displacements considered by our model.

Let $${v}_{a}\left(t;{{\varvec{p}}}_{{v}_{a}}\right)\in {\mathcal{R}}^{+}$$ be the local bone growth rate (in days^−1^) tangential to the cranial surface at a location $$a$$ in a cranial suture with coordinates $${{\varvec{x}}}_{a}$$ at age $$t$$, $${z}_{a}\left(t;{{\varvec{p}}}_{{z}_{a}}\right)$$ the velocity of the local displacement (in mm/days) observed at such location produced by the expansion of the facial and cranial base structures perpendicular to the cranial surface, and $${{\varvec{p}}}_{a}=\left\{{{\varvec{p}}}_{{v}_{a}}, {{\varvec{p}}}_{{z}_{a}}\right\}$$ the parameters defining $${v}_{a}$$ and $${z}_{a}$$ (see Table 1 for a summary of mathematical symbols). The transformed coordinates at age $$T$$ of a point on the cranial surface with initial coordinates $${\varvec{x}}$$ at age $${t}_{0}$$ caused by the bone growth ($${v}_{a})$$ and displacement ($${z}_{a}$$) at suture location $$a$$ with coordinates $${{\varvec{x}}}_{a}$$ can be written as

1 $${\boldsymbol{\Phi }}_{a}^{{t}_{0}}\left({\varvec{x}},T; {{\varvec{p}}}_{a}\right)={\varvec{x}}+{\int }_{{t}_{0}}^{T}\left[{v}_{a}\left(t;{{\varvec{p}}}_{{v}_{a}}\right)\langle {\boldsymbol{\Phi }}_{a}^{0}\left({\varvec{x}},t;{{\varvec{p}}}_{a}\right)-{\boldsymbol{\Phi }}_{a}^{0}\left({{\varvec{x}}}_{a},t;{{\varvec{p}}}_{a}\right),{{\varvec{u}}}_{a}\rangle {{\varvec{u}}}_{a}+{z}_{a}\left(t;{{\varvec{p}}}_{{z}_{a}}\right){{\varvec{y}}}_{a}\right]dt,$$

where ${{\varvec{u}}}_{a}$ is a unitary vector tangential to the cranial surface and perpendicular to the suture at ${{\varvec{x}}}_{a}$, and ${{\varvec{y}}}_{a}$ is a unitary vector perpendicular to the cranial surface at ${{\varvec{x}}}_{a}$. ${v}_{a}$ and ${z}_{a}$ are scalars representing the scaling and translation components of an affine velocity field centered at ${{\varvec{x}}}_{a}$, respectively. $\langle \cdot , \cdot \rangle$ represents the inner product operation. Note that the displacement component ${z}_{a}\left(t;{{\varvec{p}}}_{{z}_{a}}\right)$ only impacts the boundary between the calvaria and the cranial base as represented in Fig. 2a to account for the development of the lower cranial structures that are not considered in this model, and it does not model bone growth.

**Table 1:** Mathematical notations, summarized in the order of their appearance in the manuscript.

Notation	Description
*a*	Anchor index on the cranial sutures
***x***_*a*_	The Euclidean coordinate at location *a*
${v}_{a}$	Local suture growth rate tangential to the cranial surface and perpendicular to the suture at location $a$
${z}_{a}$	Velocity at location $a$ produced by the expansion of the lower cranial structures perpendicular to the cranial surface
${{\varvec{p}}}_{a}=\left\{{{\varvec{p}}}_{{v}_{a}}, {{\varvec{p}}}_{{z}_{a}}\right\}$	Vector of parameters defining ${v}_{a}$ and ${z}_{a}$
${\boldsymbol{\Phi }}_{a}^{{t}_{0}}\left({\varvec{x}},T; {{\varvec{p}}}_{a}\right)$	The transformed coordinates at age $T$ of a point on the cranial surface with initial coordinates ${\varvec{x}}$ at reference age ${t}_{0}$ caused by the expansion and displacements produced at suture location $a$ with coordinates ${{\varvec{x}}}_{a}$
${{\varvec{u}}}_{a}$	A unitary vector tangential to the cranial surface at suture anchor $a$ and perpendicular to the suture
${{\varvec{y}}}_{a}$	A unitary vector perpendicular to the cranial surface at suture anchor $a$
$\langle \cdot , \cdot \rangle$	Inner product operation
${w}_{l}^{a}\left({\varvec{x}}\right)$	Weight at coordinates ${\varvec{x}}$ associated with the affine velocity field produced at location *a* in suture $l$
${{\varvec{p}}}_{{w}_{a}}$	Parameter vector defining ${w}_{l}^{a}$
${{\varvec{I}}}_{0}\left({\varvec{m}}\right)$	The Euclidean coordinates at location ${\varvec{m}}$ in the average spherical anatomical map at birth
${\varvec{R}}\left({\varvec{\theta}}\right)$	A matrix representing a rigid spatial transformation with parameters ${\varvec{\theta}}$
${d}_{l}({\varvec{x}})$	The Euclidean distance between location ${\varvec{x}}$ and the cranial bones separated by suture $l$
*k*	Parameter controlling the smoothness of our weight functions ${w}_{l}^{a}$
${{\varvec{\Sigma}}}_{l}$	The variance matrix of the Gaussian kernel used in the weight function ${w}_{l}^{a}$
$\epsilon (t,p)$	Trace of the Green–Lagrange strain tensor at time $t$ at any point $p$ of the cranial surface
${\varvec{d}}(t,p)$	Displacement vector from birth to age $t$ at any point $p$ of the cranial surface
$\nabla$	Spatial derivative operator
$tr()$	Trace operator

As previously shown^32^, the combination of affine velocity fields centered at different locations such as the one presented in Eq. (1) can result into complex deformable spatial transformations. In our application, we aim to find a deformable spatiotemporal mapping between the observed cranial anatomy of a subject at a specific age and its future anatomical shape at a different age by combining the affine velocity fields produced by the local growth at each suture. As we previously showed^25^, different smooth and invertible weight functions can be used to model the area of influence and combine different local affine velocity fields to create a deformable diffeomorphic transformation model. Following a similar approach, we propose associating a spatial weight function with the affine velocity field produced at every suture location. Then, they can be combined to calculate the transformed coordinates of a point on the cranial surface with initial coordinates ${\varvec{x}}$ at age ${t}_{0}$ on the cranial surface to age $T$ as

2 $${{\varvec{\Phi}}}^{{t}_{0}}\left({\varvec{x}},T; {\varvec{p}}\right)={\varvec{x}}+{\int }_{{t}_{0}}^{T}{\int }_{a \in {\Omega }_{l},\forall l}{w}_{l}^{a}({\varvec{x}})\left[{v}_{a}\left(t;{{\varvec{p}}}_{{v}_{a}}\right)\langle {\boldsymbol{\Phi }}_{a}^{0}\left({\varvec{x}},t;{{\varvec{p}}}_{a}\right)-{\boldsymbol{\Phi }}_{a}^{0}\left({{\varvec{x}}}_{a},t;{{\varvec{p}}}_{a}\right),{{\varvec{u}}}_{a}\rangle {{\varvec{u}}}_{a}+{z}_{a}\left(t;{{\varvec{p}}}_{{z}_{a}}\right){{\varvec{y}}}_{a}\right]da dt,$$

where ${w}_{l}^{a}\left({\varvec{x}}\right)$ is the weight or contribution of the affine velocity field from location ${{\varvec{x}}}_{a}$ at suture $l$ at coordinates ${\varvec{x}}$, ${\Omega }_{l}$ is the spatial domain of the cranial suture $l$, and ${\varvec{p}}=\left\{{{\varvec{p}}}_{a},{{\varvec{p}}}_{{w}_{a}}\right\},\forall a\in {\Omega }_{l}$ is a vector concatenating the parameters ${{\varvec{p}}}_{{\varvec{a}}}=\left\{{{\varvec{p}}}_{{v}_{a}}, {{\varvec{p}}}_{{z}_{a}}\right\}$ of the affine velocity fields and the parameters ${{\varvec{p}}}_{{w}_{a}}$ which is associated with the weight functions ${w}_{l}^{a}$ from every suture location $a$. The implementation of this weight function is described in section "Modeling the area of suture influence". Note that, in this formulation, all weights from all suture locations are normalized so ${\int }_{a \in {\Omega }_{l},\forall l}{w}_{l}^{a}\left({\varvec{x}}\right)da=1$. Equation (2) explicitly models the transformation of any point in the cranial surface as a consequence of only sutural growth and the displacements produced by the development of the facial and cranial base structures.

### Model parameter inference

We inferred all model parameters ${\varvec{p}}$ using our cross-sectional normative CT images in Dataset A. First, we calculated the average anatomical cranial shape at birth using the aligned 2D spherical map representations of the cranial anatomy of our subjects. Then, we estimated the model parameters ${\varvec{p}}$ that minimized the difference between the predicted average anatomical development with our model and all the anatomical observations from Dataset A as:

3 $${\varvec{p}}={\mathrm{arg}}\underset{{\varvec{p}},{\varvec{\theta}}}{{\min}}\left(\frac{1}{N}\sum_{\forall s}\frac{1}{M}\sum_{\forall m}\frac{1}{{\left|\left|{{\varvec{I}}}_{s}\left({\varvec{m}}\right)\right|\right|}_{2}}||{\boldsymbol{\Phi }}^{{t}_{0}}\left({ {\varvec{I}}}_{0}\left({\varvec{m}}\right), {t}_{s};\boldsymbol{ }{\varvec{p}}\boldsymbol{ }\right)-{\varvec{R}}\left({\varvec{\theta}}\right){{\varvec{I}}}_{s}\left({\varvec{m}}\right)|{|}_{2}\right),$$

where ${{\varvec{I}}}_{0}\left({\varvec{m}}\right)$ represents the Euclidean coordinates at location ${\varvec{m}}$ in the average spherical anatomical map at birth, ${{\varvec{I}}}_{s}\left({\varvec{m}}\right)$ are the Euclidean coordinates at the same location in the anatomical map of subject $s$ with age ${t}_{s}$, $N$ is the number of subjects in Dataset A, $M$ is the number of points in our standard 2D spherical representation, and ${\varvec{R}}\left({\varvec{\theta}}\right)$ is a matrix representing a rigid spatial transformation with rotation and translation parameters ${\varvec{\theta}}$. Note that ${\varvec{R}}\left({\varvec{\theta}}\right)$ is not part of our model and it is only used during optimization to minimize the impact of potential pose standardization inaccuracies in pre-processing stages. The differences between the model and the anatomical observation from each subject are scaled by the inverse of ${\left|\left|{{\varvec{I}}}_{s}\left({\varvec{m}}\right)\right|\right|}_{2}$ to compensate for differential contribution to our objective function of subjects with different cranial size.

### Implementation

#### Modeling the area of suture influence

As indicated in Eq. (2), the influence of each suture location to the growth at any point on the cranial surface with coordinates ${\varvec{x}}$ is determined by the weight functions ${w}_{l}^{a}({\varvec{x}}), \forall a\in {\Omega }_{l},\forall l$. These functions should ensure that bone growth at suture $l$ only affects the cranial bones that this suture separates. Hence, we propose to define the area of influence of every suture using the expression:

4 $${w}_{l}\left({\varvec{x}}\right)={e}^{-k\cdot {d}_{l}({\varvec{x}})},$$

where ${d}_{l}({\varvec{x}})$ represents the Euclidean distance between ${\varvec{x}}$ and the cranial bones separated by suture $l$, and $k$ controls the smoothness of ${w}_{l}$ at their boundary. Figure 2c shows the values of ${w}_{l}$ calculated for the metopic suture. As it can be observed, this function takes a value of one at the frontal bones, which are separated by the metopic suture, and it rapidly changes to 0 outside them.

Since there may be differential bone growth at different locations of any cranial suture, we uniformly sampled each suture using control points at discretized locations ${{\varvec{x}}}_{a}\in {\Omega }_{l}$, which enabled creating spatial gradients within every suture using spatial kernels centered at those locations as shown in Fig. 2a and b. Hence, we defined the continuous spatial weight function that represents the contribution of the affine velocity field from each sampled location ${{\varvec{x}}}_{a}$ in suture $l$ to the growth observed at any point with coordinates ${\varvec{x}}$ as

5 $${w}_{l}^{a}\left({\varvec{x}}\right)={w}_{l}\left({\varvec{x}}\right){e}^{-0.5{\left({\varvec{x}}-{{\varvec{x}}}_{a}\right)}^{T}{\Sigma }_{l}\left({\varvec{x}}-{{\varvec{x}}}_{a}\right)},$$

where ${{\varvec{\Sigma}}}_{l}$ is the variance matrix of the spatial kernel. Figure 2d shows the values of ${w}_{l}^{a}\left({\varvec{x}}\right)$ for an exemplar location in the metopic suture. The parameters $k$ and ${{\varvec{\Sigma}}}_{l}$ define the exact area of influence of each cranial suture that are included in ${{\varvec{p}}}_{{w}_{a}}$ and are estimated through optimization together with the rest of parameters ${\varvec{p}}$ of our model as described next.

#### Parameter estimation and model evaluation

Since cranial expansion presents a semi-logarithmic temporal profile^21, 33–35^, we modeled the local growth rate ${v}_{a}$ of at suture location $a$ using a velocity function that can take the form of the temporal derivative of a logarithmic growth function as

6 $${v}_{a}\left(t;{{\varvec{p}}}_{{{\varvec{v}}}_{{\varvec{a}}}}\right)=\frac{1}{{p}_{{v}_{a},0}+{p}_{{v}_{a},1}t+{p}_{{v}_{a},2}{t}^{2}} ,$$

where $t$ represent age and ${{\varvec{p}}}_{{{\varvec{v}}}_{{\varvec{a}}}}=\{{p}_{{v}_{a},0},\dots ,{p}_{{v}_{a},2}\}$ are the parameters defining the growth rate. Similarly, we propose the following model for the velocity associated with the displacements produced the development of the facial and cranial base structures:

7 $${z}_{a}\left(t;{{\varvec{p}}}_{{z}_{a}}\right)=\frac{1}{{p}_{{z}_{a},0}+{p}_{{z}_{a},1}t+{p}_{{z}_{a},2}{t}^{2}},$$

where ${{\varvec{p}}}_{{{\varvec{z}}}_{{\varvec{a}}}}=\{{p}_{{z}_{a},0},\dots ,{p}_{{z}_{a},2}\}$ are the parameters of ${z}_{a}$. Note that these displacements are only modeled at the boundary of the calvaria.

To estimate all model parameters, we first discretized Eq. (2) as

8 $${{\varvec{\Phi}}}^{{{\varvec{t}}}_{0}}\left({\varvec{x}},T; {\varvec{p}}\right)={\varvec{x}}+{\sum }_{\mathrm{t}=0}^{\mathrm{T}}{\sum }_{\forall a}{w}_{l}^{a}\left({\varvec{x}}\right)\left[{v}_{a}\left(t;{{\varvec{p}}}_{{v}_{a}}\right)\langle {\boldsymbol{\Phi }}_{a}^{0}\left({\varvec{x}},t;{{\varvec{p}}}_{a}\right)-{\boldsymbol{\Phi }}_{a}^{0}\left({{\varvec{x}}}_{a},t;{{\varvec{p}}}_{a}\right),{{\varvec{u}}}_{a}\rangle {{\varvec{u}}}_{a}+{z}_{a}\left(t;{{\varvec{p}}}_{{z}_{a}}\right){{\varvec{y}}}_{a}\right]\Delta t$$

where $\Delta t$ is the temporal discretization interval, and $T$ the time span of our dataset. In our implementation, we used a discretization interval of 5 days because it was shown that temporal interpolation at finer discretization intervals do not significantly improve prediction accuracies^22^. Finally, we used the implementation of the adaptive moment estimation (Adam) gradient descent algorithm in PyTorch 3.10.4 with an initial learning rate of 0.01 to estimate the model parameters using Eq. (3).

After estimating the model parameters, we calculated the fitting error of the model to the normative cross-sectional Dataset A as the local Euclidean distance between the trained average model and their observed anatomy at each point. We also evaluated its predictive performance using the longitudinal studies of normative subjects in Dataset B. Specifically, we first segmented the cranial bones from the first available CT image of each subject, and we calculated the personalized suture growth and displacement vectors (${{\varvec{u}}}_{a}$ and ${{\varvec{y}}}_{a}$ in Eq. (8)) from the segmented cranial anatomies. Since all spherical maps were aligned at the cranial sutures and represented in a standard normalized space, the weight functions ${w}_{l}^{a}$ calculated in the spatial domain of the 2D spherical maps do not change between subjects. We then used the previously inferred suture growth parameters to predict growth at the time of the second available CT image for each subject in Dataset B using Eq. (8). Finally, we quantified the predictive error as the distance between the observed and the predicted anatomies.

We also evaluated the accuracy of the model simulating craniosynostosis using Dataset C. Specifically, we used Eq. (8) to predict growth from birth to the age of every patient in Dataset C using the learned model parameters but setting the suture growth rates ${v}_{a}$ within the fused sutures to zero to simulate fusion. We compared the predicted average shape in presence of suture fusion with the observed anatomies to quantify the simulation error.

Finally, we used the longitudinal image pairs in Dataset D to evaluate our model predicting patient-specific growth in presence of suture fusion. We used the observed anatomies in the first study of each pair to calculate the vectors ${{\varvec{u}}}_{a}$ and ${{\varvec{y}}}_{a}$. Then, we used Eq. (8) and the trained model parameters to predict the anatomy at the time of acquisition of their latter available image but setting the growth rates of their fused suture to zero. The Mann-Whiney U-test was used to compare the performance of the model between the different datasets with a significance level of 0.05.

## Results

### Model fitting and prediction of normative subjects

**Figure 3:**
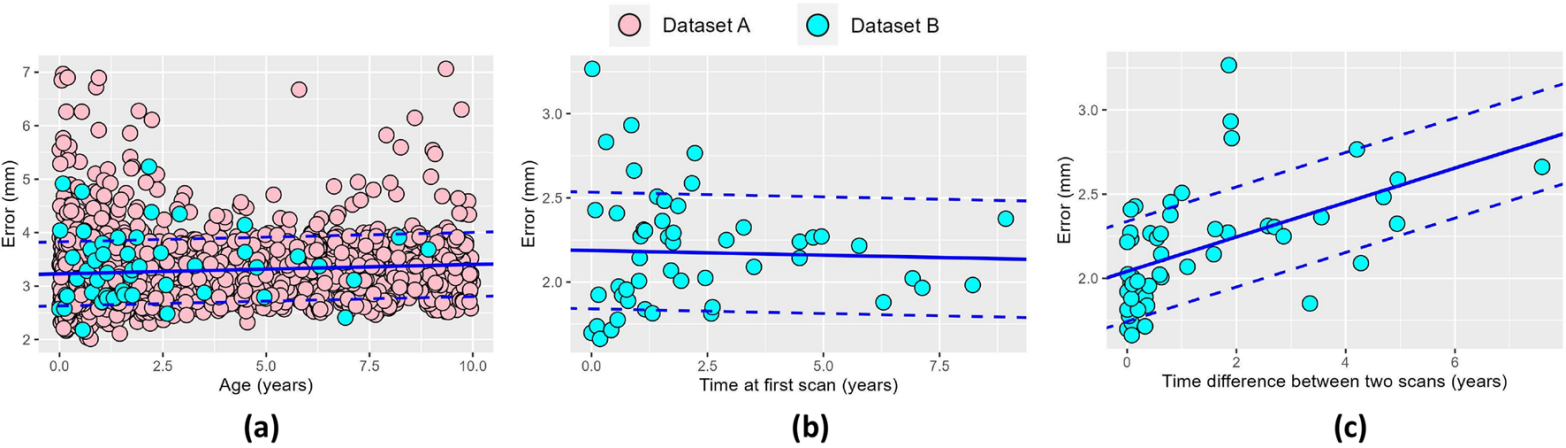
Accuracy of our model on normative subjects. (**a**) shows the distance between our average normative model and the normative cranial anatomies from Datasets A and B. (**b**) shows the prediction error evaluated on the longitudinal images of Dataset B as a function of patient age in the first available study. (**c**) shows the prediction error evaluated on the longitudinal images of Dataset B as a function of the time between the two available longitudinal studies. A linear regression of the error is shown in each figure as solid and dotted blue lines for the average and the range of one standard deviation around the mean, respectively.

**Figure 4:**
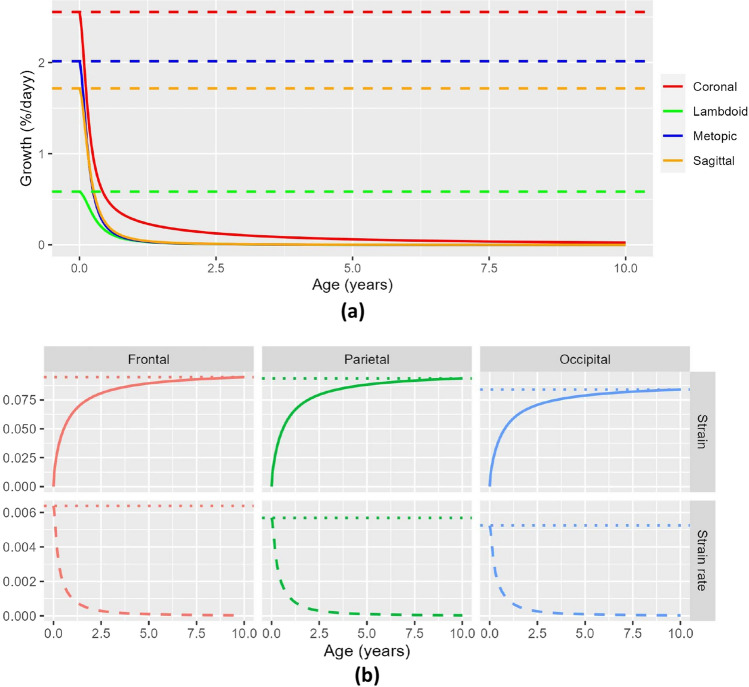
Average suture and bone growth as a function of age. (**a**) Represents the suture growth rates inferred from Dataset A. (**b**) Represents the average strain and strain rates in each cranial bone.

**Figure 5:**
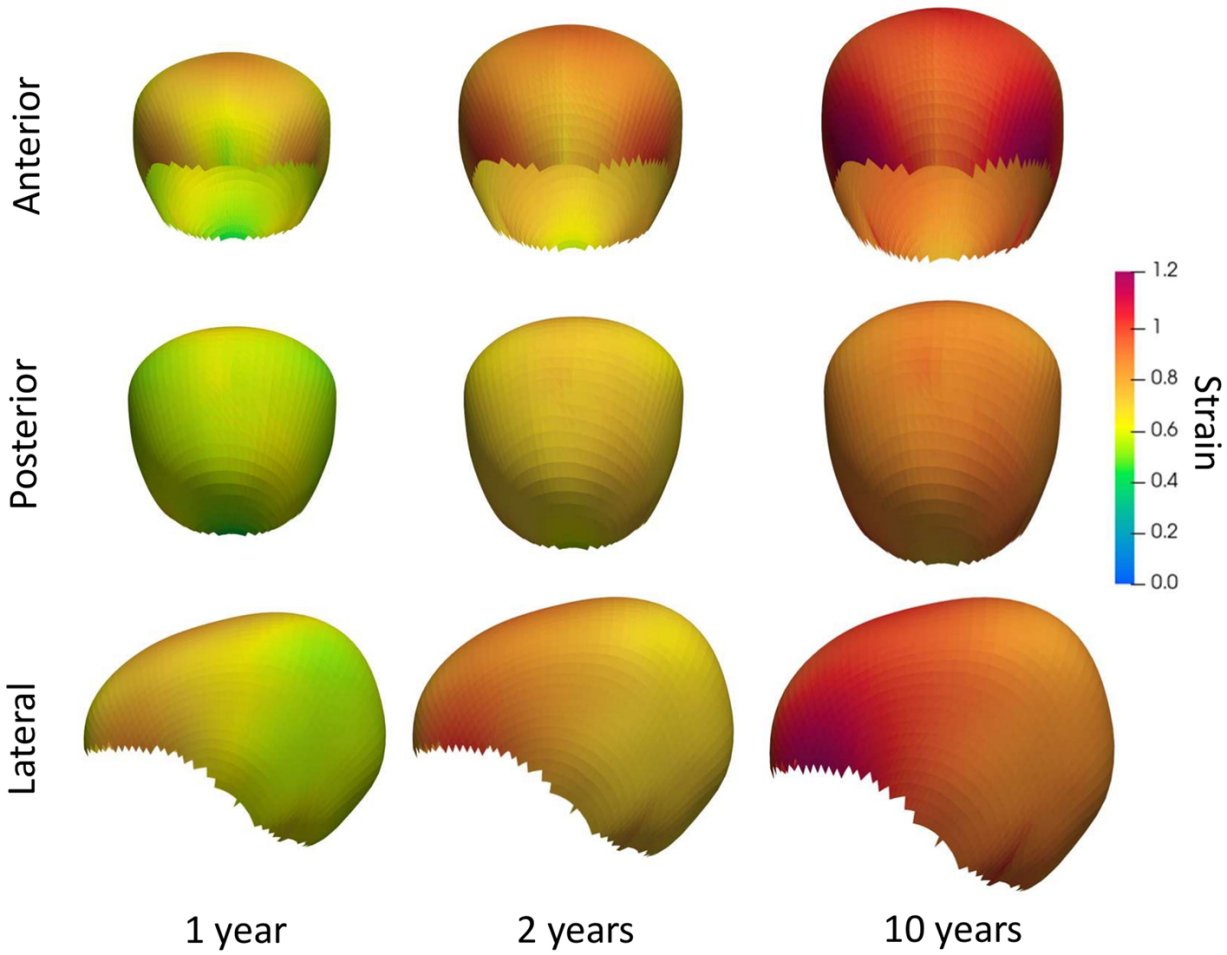
Shape development generated using the trained model, color-coded in strain values. The first, second, third rows represent the anterior, the posterior, and the lateral view, respectively. The first, second, and third columns represent the cranial shapes at 1 year, 2 years, and 10 years of age, respectively.

We trained our model using Dataset A, where we achieved an average fitting error of 3.28 ± 0.60 mm as shown in Fig. 3a. Figure 4a shows the average growth rates ${v}_{a}\left(t;{{\varvec{p}}}_{{{\varvec{v}}}_{{\varvec{a}}}}\right)$ inferred at every cranial suture that explain the cross-sectional observations in Dataset A. In addition, we calculated the trace of the Green–Lagrange strain at each point on the average cranial surface of our trained model to quantify differential local growth patterns across different areas of the cranium as

9 $$\epsilon \left(t,p\right)=tr\left(\frac{\nabla {\varvec{d}}\left(t,p\right)+\nabla {{\varvec{d}}\left(t,p\right)}^{T}+\nabla {\varvec{d}}\left(t,p\right)\nabla {{\varvec{d}}\left(t,p\right)}^{T}}{2}\right),$$

were ${\varvec{d}}$ represents the displacement vector from birth to age $t$ at any point $p$, $\nabla$ is the spatial derivative operator, and $tr$ represents the trace of the tensor. Figure 4b shows the average value of $\epsilon$ and its temporal derivative (strain rate) at every cranial bone. Figure 5 shows the strain distributions in the calvarium at 1, 2, and 10 years of age.

We tested the predictive accuracy of our model using Dataset B. The average distance between the segmented cranial anatomies from the first images in this independent dataset and our cranial model was 3.36 ± 0.65 mm, which was similar to the fitting error to the training dataset (p = 0.27 evaluated using a Mann–Whitney U test) as shown in Fig. 3a. Then, we used the previously inferred suture growth parameters to predict growth at the time of the second available CT image for each subject in Dataset B as described in section “Parameter estimation and model evaluation”. We obtained an average predictive error of 2.17 ± 0.34 mm, which was significantly lower than the average fitting error to the first CT image (p < 0.001 using Mann–Whitney U test). Figure 3b and c shows linear regressions of the prediction error as a function of age and of the time between longitudinal images, respectively. The predictive error for normative growth was significantly associated with the time between studies (p < 0.001), but not with the age at the first study (p = 0.8).

### Prediction of craniosynostosis

Modeling cranial bone development as a function of sutural growth allows modifying the inferred sutural growth rates to simulate cranial suture pathology. Benefitting from this explicit model representation, we evaluated its ability to predict growth in presence of single suture craniosynostosis using Datasets C and D as previously described. We did not incorporate the lambdoid sutures because of lack of data for this rare type of craniosynostosis^36–38^.

**Figure 6:**
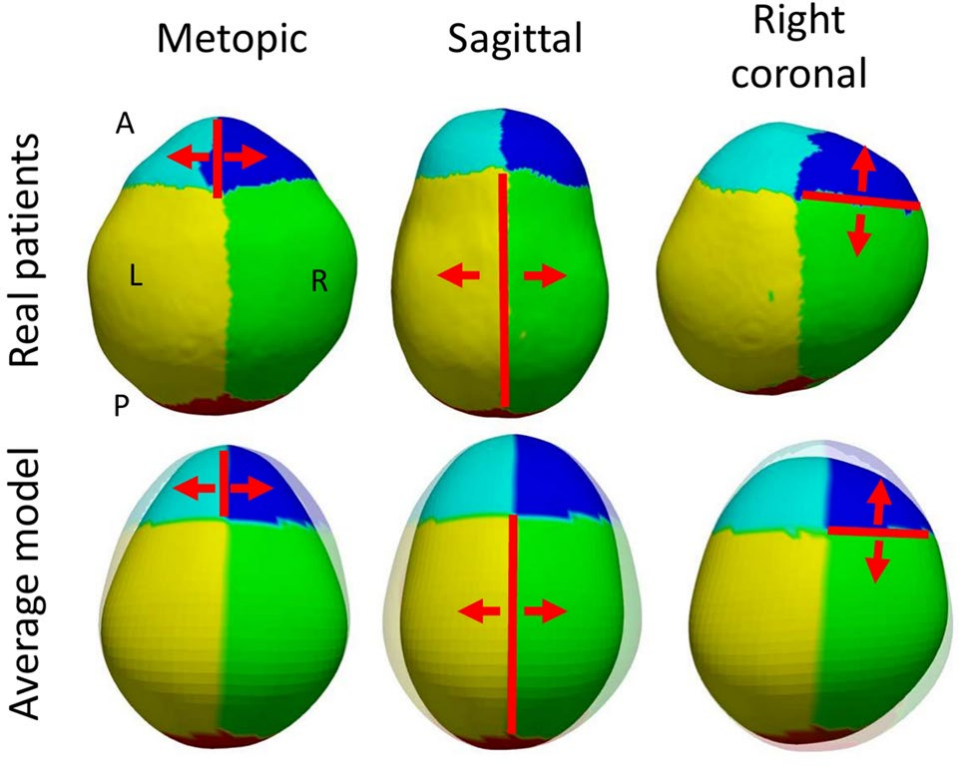
Simulation of the average abnormal phenotypes of single suture craniosynostosis using the trained model. The top row shows a superior view of the cranial anatomy of three patients with metopic (female, age 10 months), sagittal (male, age 13 months) and single right coronal (female, age 8 months) craniosynostosis. The bottom row shows average simulated models of single suture craniosynostosis between birth and the age of each patient, with shaded shapes representing the average normative shape for the age and sex of each patient. Red lines show the fused sutures for reference, and red arrows show the direction of constrained growth. A, P, L and R represent anterior, posterior, left and right, respectively.

Using the method described in section “Parameter estimation and model evaluation”, we compared the predicted average shape in presence of suture fusion with the observed anatomies in Dataset C and we obtained an average difference of 3.45 ± 1.22 mm (p = 0.29 compared to the fitting errors to Dataset A using Mann–Whitney U test). Specifically, we obtained errors of 2.60 ± 0.93 mm, 3.13 ± 1.26 mm and 3.77 ± 1.15 mm for patients with metopic, unicoronal and sagittal craniosynostosis, respectively. Interestingly, these errors were significantly lower than the fitting error to Dataset A for patients with metopic (p < 0.001) and unicoronal craniosynostosis (p < 0.001), and significantly higher for patients with sagittal craniosynostosis (p < 0.001). Despite variable accuracy results, we show qualitatively in Fig. 6 how our model can reproduce the expected typical abnormal phenotypes associated with the individual fusion of the metopic, coronal or sagittal sutures.

**Figure 7:**
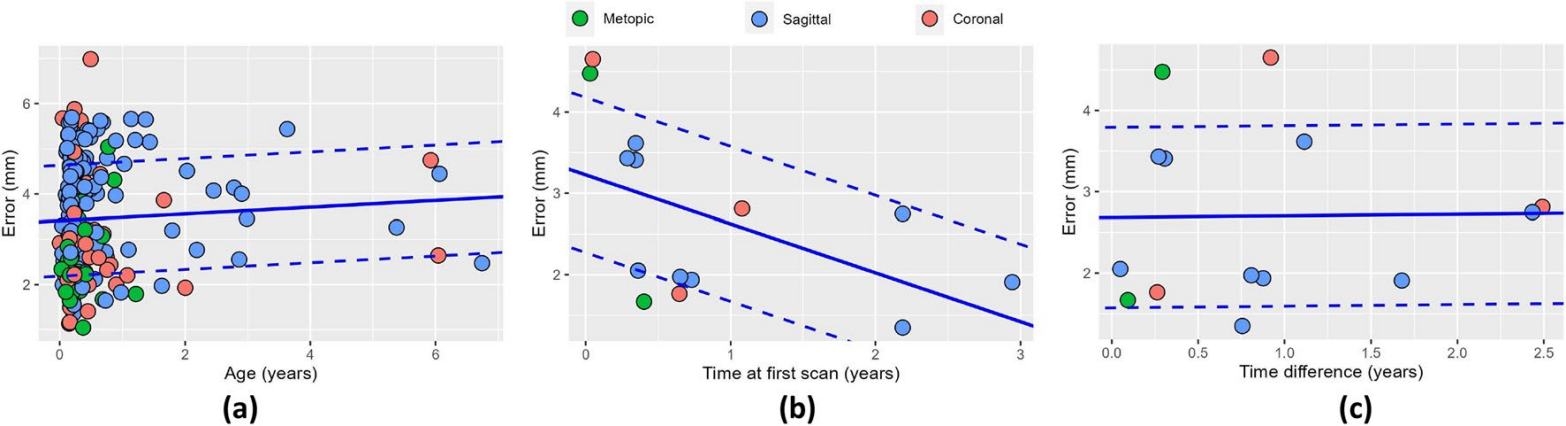
Accuracy of our model on patients with craniosynostosis. (**a**) shows the distance between our average model of single suture craniosynostosis and the cranial anatomies from Dataset C. (**b**) shows the prediction error evaluated on the longitudinal images of Dataset D as a function of patient age in the first available study. (**c**) shows the prediction error evaluated on the longitudinal images of Dataset D as a function of the time between the two available longitudinal studies. A linear regression of the error is shown in each figure as solid and dotted blue lines for the mean error and the range of one standard deviation around the mean, respectively.

We used the longitudinal image pairs in Dataset D to evaluate our model predicting patient-specific growth in presence of suture fusion. We achieved an average prediction error of 2.70 ± 1.07 mm (3.07 ± 1.98 mm, 3.08 ± 1.46 mm and 2.49 ± 0.83 mm for patients with metopic, unicoronal and sagittal craniosynostosis, respectively), which was significantly lower than the fitting error to both Datasets A (p = 0.02 using Mann–Whitney U test) and C (p = 0.02) and not significantly different from the normative predictive error evaluated using Dataset B (p = 0.31). Figure 7a shows the fitting error of our model in Dataset C. Figure 7b and c shows linear regressions of the predictive errors as functions of the time at the first study and the time between longitudinal studies in Dataset D, respectively. The predictive errors for patients with craniosynostosis were not significantly associated with either patient age at the first study (p = 0.06) or the time between two studies (p = 0.38).

## Discussion

Inspired by previous works on locally affine image registration^25^, we presented a novel data-driven diffeomorphic cranial bone development model between birth and 10 years of age designed to explain cranial growth by bone expansion at the sutures. We proposed a local weighting scheme that can produce deformable cranial transformations explained only by bone growth at the cranial sutures and accounting for displacements produced by the development of the facial and cranial base structures. As it can be observed in Fig. 2c, d, the locality of our transformation model achieved through our proposed weighting scheme ensures that the growth observed at every cranial suture affects the expansion of its surrounding cranial bone plates. In addition, we introduced directional constraints to ensure that growth in the cranial sutures occurs perpendicular to them^3^, which is essential to inferring realistic and explainable bone development patterns. Our model overcomes the main limitation of existing simulation (based on assumptions about growth rates) and statistical methods (hardly explainable and not useful to simulate pathology). Unlike existing longitudinal reports on population statistics at discrete time points, our model offers a predictive approach that can anticipate individualized local cranial growth in the absence or presence of suture growth constraints. The model parameters defining both the local bone growth rates at every suture and the area of influence of each suture were inferred statistically from real observations, and they provide an explicit quantitative explanation of cranial development as a consequence of sutural growth as shown in Fig. 4. To our knowledge, this is the first explicit data-driven quantitative reference of sutural growth inferred from large datasets of real human data.

We evaluated the personalized predictive accuracy of our model using an independent normative longitudinal dataset and we obtained state-of-the-art performance^22^. As shown in Fig. 4a, our model inferred a faster growth in the coronal sutures during the first months of life compared to the rest of the sutures. This can explain the observed faster antero-posterior bone expansion (perpendicular to the coronal sutures) compared to the lateral direction in previous works^22^. A similar anterior–posterior pattern is also shown in Fig. 4b, where a faster expansion of the frontal bones is observed compared to the parietal bones, which also grow faster than the occipital bone. Such anterior–posterior pattern is consistent with previous reports^22^. As shown in the local deformation patterns in Fig. 5, higher rates of deformation are similarly observed around the coronal sutures compared to the rest of the areas. Our model produces average normative head shapes with cephalic index (the ratio between the lateral and the antero-posterior cranial distances) decreasing from 81.71% at birth to 77.54% at 2 years of age, which aligns with previous clinical reports on a decreasing cephalic index during early childhood^3, 21^.

We also evaluated if our model could be used to simulate craniosynostosis. As shown in Fig. 6, our model realistically simulates the expected abnormal phenotypes of patients with single fusion of the metopic (trigonocephaly), sagittal (scaphocephaly) and coronal (anterior plagiocephaly) sutures. We quantified its accuracy reproducing the abnormal cranial shapes in the presence of craniosynostosis by comparing our simulations with a cross-sectional dataset of patients (Dataset C). We obtained differences between the average model and the observed anatomies that were lower than the fitting errors of our model to the normative dataset for both patients with metopic and patients with unicoronal craniosynostosis. However, these errors were higher for patients with sagittal craniosynostosis. We also quantified the ability of our model to predict personalized growth in longitudinal dataset of patients with craniosynostosis (Dataset D). We obtained predictive errors that were lower than the difference between our average predictions of craniosynostosis and the observed cross-sectional phenotypes in Dataset C. Higher errors obtained when comparing our simulation of craniosynostosis at birth with the cross-sectional dataset C may be related to our assumption that sutures were fused at the time of birth for all patients in this dataset. Unfortunately, it is currently not possible to know the exact timeline of suture fusion for patients with craniosynostosis. However, once suture fusion is confirmed in a CT image study, our method enables making predictions of development with accuracies that are not significantly different to the predictions made for normative subjects (see experiments with longitudinal Datasets B and D). Future work could include further exploration of potential timelines of suture fusion for specific patients using the available model, which could provide insight to elucidate potential causes of suture fusion that are unknown for more than 85% of these patients^9, 39^.

**Figure 8:**
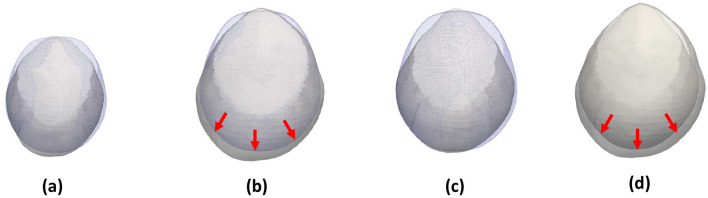
Superior view of the growth prediction of a patient with metopic craniosynostosis in Dataset D. (**a**) shows the real observation at the time of the first image study (11 days) compared with the average normative shape, represented as a transparent overlay. (**b**) shows the real observation of the same patient at the time of the second study (118 days) compared with the average normative shape. (**c**) shows the comparison between the prediction generated from our model and the average normative shape. (**d**) shows the comparison between the real observation and the prediction. Average shapes are represented using surfaces with blue edges. Red arrows represent the area of compensatory growth.

The diverse performance of our model in patients with craniosynostosis may also be related to the compensatory volume mechanisms observed in patients with craniosynostosis parallel to the fused sutures^3^. This effect is demonstrated in Fig. 8, where we show a comparison between the observed anatomies of a patient with metopic craniosynostosis, our average normative shape at the same age, and the personalized predictions from our model at different longitudinal observations. As it can be observed, while our model can simulate the abnormal constrained growth in the anterior area of the cranium, the patient presents a posterior compensatory volume overdevelopment produced by an increased pressure from the growing brain^3^. Our model only presented a lower accuracy than the fitting error for our group of patients with sagittal craniosynostosis. This may be explained because the parietal bones that are separated by the sagittal suture have larger surfaces, which may translate into a larger volumetric constraint that may also produce larger volume overdevelopment in other areas. Unfortunately, the quantitative understanding of the complex relationships between the exact time of suture fusion, the local increased pressure from a growing brain and the compensatory overdevelopment that occurs as a consequence of it is still very limited. Our model constitutes an important step towards quantitatively understanding cranial growth at the sutures in children. Future work includes the study of volume compensation patterns in patients with craniosynostosis and how they relate to specific local growth constraints.

Prior work has successfully modeled cranial growth and/or created personalized temporal growth predictions for healthy subjects using statistical data^21, 22^. Other studies have also successfully identified abnormal phenotypes associated with craniosynostosis for screening and diagnostic purposes^29, 40^. However, previous data-driven studies could not quantify cranial growth at the sutures and hence, they could not make temporal predictions in patients with suture growth anomalies. The current work presents the first explicit statistical reference of sutural growth in humans inferred from clinical observations to study suture growth anomalies, establishing a statistical framework to understand how suture develops for future investigations. Moreover, our study constitutes an important step towards enabling personalized temporal predictions of development in patients with cranial suture pathology, which is important to study how cranial growth restrictions may affect local and global cranial and brain volume development. This is essential to investigate the impact of surgical timing on cranial and brain growth in patients with craniosynostosis, which has the potential to be integrated into clinical practice to better inform treatment planning and improve outcomes. Our model will also provide a necessary tool to study the abnormal phenotypes of patients with craniosynostosis in the context of temporal sutural growth. Finally, our work shows both quantitatively and qualitatively the importance of understanding the volume compensatory mechanisms in the cranium, which will be subject of future work to understand the coupled developmental mechanisms between the cranium and the brain.

## Data Availability

The data used in this study corresponds to head CT images of pediatric patients that include private health information. Hence, these data cannot be made public due to HIPAA regulations. As part of a previous study, a generative normative model^21^ built from this dataset has been made available in https://github.com/cuMIP/normativeCranialGrowth. The sutural growth model presented in this study is available in https://github.com/cuMIP/cranialSutureGrowth.
